# Relationship of Perfluorooctanoic Acid Exposure to Pregnancy Outcome Based on Birth Records in the Mid-Ohio Valley

**DOI:** 10.1289/ehp.1104752

**Published:** 2012-03-26

**Authors:** David A. Savitz, Cheryl R. Stein, Beth Elston, Gregory A. Wellenius, Scott M. Bartell, Hyeong-Moo Shin, Veronica M. Vieira, Tony Fletcher

**Affiliations:** 1 Department of Epidemiology, Brown University, Providence, Rhode Island, USA; 2 Department of Preventive Medicine, Mount Sinai School of Medicine, New York, New York, USA; 3 Program in Public Health, and; 4 School of Social Ecology, University of California, Irvine, Irvine, California, USA; 5 Department of Environmental Health, Boston University School of Public Health, Boston, Massachusetts, USA; 6 London School of Hygiene and Tropical Medicine, London, United Kingdom

**Keywords:** fetal growth restriction, perfluorooctanoic acid, pregnancy, pregnancy-induced hypertension, preterm birth, stillbirth

## Abstract

Background: Perfluorooctanoic acid (PFOA) is a potential cause of adverse pregnancy outcomes, but previous studies have been limited by low exposures and small study size.

Objectives: Using birth certificate information, we examined the relation between estimated PFOA exposure and birth outcomes in an area of West Virginia and Ohio whose drinking water was contaminated by a chemical plant.

Methods: Births in the study area from 1990 through 2004 were examined to generate case groups of stillbirth (*n* = 106), pregnancy-induced hypertension (*n* = 224), preterm birth (*n* = 3,613), term low birth weight (*n* = 918), term small-for-gestational-age (SGA) (*n* = 353), and a continuous measure of birth weight among a sample of term births (*n* = 4,534). A 10% sample of term births ≥ 2,500 g were selected as a source of controls (*n* = 3,616). Historical estimates of serum PFOA were derived from a previously developed fate and transport model. In a second study, we examined 4,547 area births linked to a survey with residential history data.

Results: In the analysis based only on birth records, we found no consistent evidence of an association between estimated PFOA exposure and stillbirth, pregnancy-induced hypertension, preterm birth, or indices of fetal growth. In the analysis of birth records linked to the survey, PFOA was unrelated to pregnancy-induced hypertension or preterm birth but showed some suggestion of an association with early preterm birth. Measures of growth restriction showed weak and inconsistent associations with PFOA.

Conclusions: Based on the analysis using the health survey, these results provide little support for an effect of PFOA exposure on most pregnancy outcomes, except for early preterm birth and possibly fetal growth restriction.

Adverse effects of perfluorooctanoic acid (PFOA) and related perfluoroalkyl acids on pregnancy and development have been suggested based on toxicology (Lau et al. 2007) and a small but growing epidemiologic literature (Steenland et al. 2010). The strongest support from laboratory studies suggests the potential for reductions in fetal growth (Lau et al. 2007). Epidemiologic findings on measures of reduced growth are mixed (Apelberg et al. 2007; Fei et al. 2007; Hamm et al. 2010; Nolan et al. 2009; Washino et al. 2009), but generally consistent with a small decrement in birth weight associated with higher levels of serum PFOA. Duration of gestation has not been related to PFOA in these same studies (Apelberg et al. 2007; Fei et al. 2007; Hamm et al. 2010). More extreme, clinically consequential end points of fetal growth restriction and preterm birth have been examined less extensively and often with limited statistical power; none of the human studies support an association (Fei et al. 2007; Hamm et al. 2010; Nolan et al. 2009). Given ubiquitous exposure, suggestive toxicologic data, and limited epidemiologic research, continued examination of the potential association between PFOA and pregnancy outcomes is warranted.

A large population in Ohio and West Virginia has been exposed to PFOA predominantly through industrial contamination of drinking-water supplies (Frisbee et al. 2009). Within this population, PFOA exposure varies considerably across time and place because of different levels of PFOA contamination in the drinking water, depending on the year and the water district, enabling accurate estimation of serum levels by linking residential histories and historical drinking-water concentrations. The resulting range of PFOA levels are well above background exposure (Steenland et al. 2009), which may allow for a more informative assessment of the impact of PFOA exposure on birth outcomes.

For participants in the C8 Health Project—a survey conducted in the contaminated region (Frisbee et al. 2009)—we previously analyzed a small number of births using serum PFOA measurements (Stein et al. 2009) and a much larger number using modeling to assign estimated serum PFOA levels (Savitz et al. 2012). In this report, we used two approaches to evaluate births in the region. The first provides comprehensive coverage of the study area, but is subject to some exposure misclassification. The second sacrifices some of the sample size, but has improved exposure measurement. Both studies rely on birth certificates for assessment of health end points and use the same environmental/pharmacokinetic models to estimate PFOA exposure.

## Study I: PFOA and Pregnancy Outcome Based on Birth Records

### Methods

The study area in the mid-Ohio Valley was chosen to include PFOA-contaminated public water utilities in Mason and Wood counties in West Virginia and in Athens, Meigs, and Washington counties in Ohio, where levels ranged from near U.S. averages to orders of magnitude above typical levels (Frisbee et al. 2009). We obtained computerized live birth records with geographic identifiers from the Ohio and West Virginia health departments, and fetal death records from West Virginia only (unpublished data). We restricted the analysis to pregnancies that occurred from 1990 through 2004 because that is the period when the contrasts were highest between exposures resulting from contaminant releases and background sources (Shin et al. 2011a).

Because of the expense involved with refining address information and geocoding maternal residences, which is the basis for assigning exposure, we used case–control sampling to enhance precision for some of the birth outcomes of interest. We restricted eligibility to singleton births and excluded the small proportion (3%) of nonwhite births because there were too few to analyze separately. Comprehensively identified case groups consisted of stillbirths (fetal death ≥ 20 weeks completed gestation), preterm births (< 37 weeks completed gestation), and term low birth weight births (≥ 37 weeks completed gestation and birth weight < 2,500 g). A 10% sample of term births ≥ 2,500 g (*n* = 3,616) was selected and geocoded for assigning PFOA exposure.

To efficiently select control groups and expand the range of birth outcomes, we first constructed a stratified 10% random sample of the entire population of live births by combining the 10% sample of all term live births ≥ 2500 g with a 10% random sample of the comprehensively identified preterm cases and term low birth weight cases (the case groups described above) (*n* = 4063). Pregnancy-induced hypertension cases, term small-for-gestational-age (SGA) cases, and term birth weight births were identified exclusively from the 10% random sample of births. Control groups for each of the outcomes were derived as noncase subsets from the 10% random sample of the entire population of live births.

We conducted a series of case–control analyses: *a*) stillbirths (*n* = 106) compared with West Virginia live births (*n* = 1,844) because stillbirth information was available only from West Virginia; *b*) pregnancy-induced hypertension cases (*n* = 224) compared with births without pregnancy-induced hypertension (*n* = 3,828); *c*) preterm birth cases < 37 weeks (*n* = 3,613) and < 32 weeks (*n* = 491) corresponding to all preterm and very preterm births, respectively, compared with term births (*n* = 3,695), with assignment based on clinical assessment of gestational age; *d*) term low birth weight cases (*n* = 918) compared with term births ≥ 2,500 g (*n* = 3,616); and *e*) term SGA cases [births < 10th percentile by gestational age and sex (Oken et al. 2003)] (*n* = 353) compared with term appropriate-for-gestational-age births [births between the 10th and 90th percentiles, inclusive, by gestational age and sex (Oken et al. 2003)] (*n* = 2,990). In addition, we analyzed a continuous measure of birth weight among term births (*n* = 4,534).

The exposure metric used in the analysis is modeled serum PFOA concentration of the mother estimated for the early pregnancy period. The methods for estimating individual serum PFOA concentrations are described in detail elsewhere (Shin et al. 2011a, 2011b). Briefly, information on plant operations and chemical releases was combined with environmental characteristics of the region through a series of linked models to estimate air and water concentrations of PFOA from 1951 through 2008 (Shin et al. 2011a). Geocodes for birth residences were used to determine whether drinking water in participants’ homes was supplied by public water sources or private wells. When addresses could be geocoded only at the ZIP code level, an average exposure measure was calculated based on the proportion of the ZIP code population that was supplied by contaminated drinking water. With these estimated environmental levels of PFOA based on the geocoded maternal address, individual maternal serum levels for each pregnancy were estimated using age- and sex-based pharmacokinetic modeling with standard assumptions about water intake, body weights, and a PFOA half-life (Shin et al. 2011b).

Because we had only the residence listed on the birth certificate for the mother—not a residential history—and serum levels at a given time depend on exposure history, we assumed that she had lived in this location for the previous 6 years, which would account for approximately 63% of her serum concentration given a stable exposure rate and an estimated serum half-life of PFOA of 3.5 years (Olsen et al. 2007). Moving within the same public water supply district would have little or no impact on the estimated serum values, but moving across water districts or especially from more distant locations would lead to exposure misclassification.

We were able to geocode the maternal address listed on the birth certificate to the street level for 66% of births and to the ZIP code level for the remaining 34%, with little variation in these proportions by case or control status. We found a high correlation between the exposure estimate using street level geocode and ZIP code averages (*r* = 0.84), indicating modest loss of information from lack of street level assignment. To make optimal use of the available data, we used the ZIP code average exposure and other information from the birth certificate (maternal education, maternal age, parity, tobacco use, exposure year, residential state) to impute a new exposure estimate for the pregnancies with ZIP code geocodes only (*n* = 2,780). We used multiple imputation to generate 20 replications to obtain accurate information on the variability in the imputed values (Graham et al. 2007). Markov Chain Monte Carlo imputation was used with 200 burn-in iterations and 100 iterations between each imputation. The parameter estimates and standard errors determined from each imputed data set were pooled into a final parameter estimate and variance (Rubin 1987). Missing covariate data on maternal age (*n* = 2), maternal education (*n* = 61), parity (*n* = 6), and tobacco use (*n* = 64) were also imputed. The primary analyses incorporate the exposure estimates for the street-level geocode (66%) and the imputed exposure estimate when only ZIP code geocodes could be assigned (34%). In sensitivity analyses we considered the impact of restricting to births with exposure estimates based on street-level geocodes only and no missing covariate data.

Estimated serum PFOA concentrations were generated by calendar year, with the relevant exposure year for each pregnancy considered to be month 3 of gestation for stillbirths and month 4 for live births. The estimated serum PFOA level at the time of pregnancy was skewed, so we considered serum PFOA estimates as a continuous log-transformed measure, a continuous untransformed measure, and in quintiles of exposure. We aggregated the lowest two quintiles as the reference category for analysis because those quintiles covered a modest range typical of U.S. serum levels and our exposure estimates are best able to differentiate individual exposure at higher levels resulting from consumption of locally contaminated water.

We adjusted all analyses for maternal age (splines with 3 degrees of freedom), education (< 12, 12, 13–15, ≥ 16 years), parity (0, 1, ≥ 2), tobacco use (smoker, nonsmoker), exposure year (splines with 4 degrees of freedom), and state of residence (Ohio, West Virginia). In addition, we included gestational age (37, 38, 39, 40, 41, ≥ 42 completed weeks) as a covariate in the analysis of birth weight among term births.

For the binary outcomes, we used logistic regression to estimate the odds ratio (OR) and 95% confidence interval (CI) associated with PFOA exposure, using the log-transformed value to generate ORs for an interquartile shift from the 25th to 75th percentile, and the untransformed variable used to generate ORs per 100-ng/mL shift and for quintiles (comparing the 3rd, 4th, and 5th with the aggregated 1st and 2nd quintiles). Both crude and adjusted effect estimates were generated. We used multiple linear regression to estimate the association between PFOA and birth weight, using the same measures of estimated PFOA exposure described for the categorical outcomes. All statistical analyses were conducted using SAS version 9.2 (SAS Institute Inc., Cary, NC) and R version 2.12.1 (R Project for Statistical Computing, Vienna, Austria).

### Results

Births were nearly evenly divided by calendar period and by state (Table 1). Estimated exposure to PFOA tended to be higher for births that were more recent, among older mothers, more highly educated mothers, nonsmokers, and West Virginia residents (Table 1).

**Table 1:** Study I: PFOA and pregnancy outcome based on birth records: characteristics of study population by pregnancy outcome, Mid-Ohio Valley, 1990–2004 [n (%)].

Maternal characteristic	Stillbirth	PIH	Preterm < 37 weeks	Term LBW	SGA	All births	Estimated PFOA among all births (ng/mL) median (25th, 75th percentiles)
Total		106		224		3,613		918		353		8,253		7.7 (4.9, 17.2)
Exposure year														
1990–1994		28 (26)		51 (23)		1,068 (30)		295 (32)		117 (33)		2,685 (33)		7.0 (4.5, 17.7)
1995–1999		49 (46)		84 (38)		1,170 (32)		312 (34)		124 (35)		2,776 (34)		7.9 (5.0, 16.4)
2000–2004		29 (27)		89 (40)		1,375 (38)		311 (34)		112 (32)		2,792 (34)		8.2 (5.0, 17.6)
State of residence														
WV		88 (83)		111 (50)		1,470 (41)		401 (44)		157 (44)		3,610 (44)		10.2 (5.3, 19.8)
OH		18 (17)a		113 (50)		2,143 (59)		517 (56)		196 (56)		4,643 (56)		6.3 (4.6, 13.4)
Age (years)														
< 20		18 (17)		29 (13)		602 (17)		186 (20)		75 (21)		1,330 (16)		6.7 (4.7, 13.5)
20–24		39 (37)		67 (30)		1,187 (33)		321 (35)		138 (39)		2,743 (33)		7.2 (4.9, 16.4)
25–29		23 (22)		69 (31)		949 (26)		209 (23)		77 (22)		2,185 (26)		8.4 (4.9, 18.9)
30–34		17 (16)		44 (20)		572 (16)		128 (14)		46 (13)		1,346 (16)		8.5 (4.9, 19.8)
≥ 35		9 (8)		15 (7)		303 (8)		74 (8)		17 (5)		649 (8)		8.4 (4.9, 19.7)
Education (years)														
< 12		21 (20)		24 (11)		772 (21)		273 (30)		113 (32)		1,701 (21)		6.9 (4.8, 14.3)
12		51 (48)		94 (42)		1,518 (42)		389 (42)		134 (38)		3,435 (42)		7.5 (4.9, 17.3)
13–15		24 (23)		67 (30)		865 (24)		167 (18)		68 (19)		1,996 (24)		8.3 (4.9, 19.8)
≥ 16		10 (9)		39 (17)		458 (13)		89 (10)		38 (11)		1,121 (14)		8.3 (5.0, 17.2)
Parity														
0		49 (46)		137 (61)		1,710 (47)		457 (50)		178 (50)		3,801 (46)		7.6 (4.9, 16.7)
1		29 (27)		46 (21)		1,104 (31)		271 (30)		102 (29)		2,668 (32)		7.7 (4.9, 17.1)
≥ 2		28 (26)		41 (18)		799 (22)		190 (21)		73 (21)		1,784 (22)		7.6 (4.8, 18.2)
Smoking status														
Smoker		27 (25)		32 (14)		1,039 (29)		430 (47)		167 (47)		2,364 (29)		7.0 (4.8, 15.1)
Nonsmoker		79 (75)		192 (86)		2,574 (71)		488 (53)		186 (53)		5,889 (71)		8.0 (4.9, 18.1)
Abbreviations: PIH, Pregnancy-induced hypertension; LBW, low birth weight. aSome Ohio residents delivered stillbirths in West Virginia.														

Stillbirth was unrelated to PFOA exposure based on either continuous or categorical exposure measures (Table 2). Although there was some suggestion of an increased risk based on the untransformed measure (OR = 1.20 per 100 ng/mL; 95% CI: 0.86, 1.68), the lack of gradient across quintiles does not support an association. Pregnancy-induced hypertension was unrelated to PFOA exposure regardless of metric (Table 2). Preterm birth was not associated with PFOA (Table 3).

**Table 2:** Study I: PFOA and pregnancy outcome based on birth records: association of PFOA with stillbirth and pregnancy-induced hypertension, Mid-Ohio Valley, 1990–2004.

Stillbirth							Pregnancy-induced hypertension						
Estimated PFOA	Live births^a^ (n)	Cases (n)	Crude OR	Adjusted^b^ OR (95% CI)	Live births (n)	Cases (n)	Crude OR	Adjusted^b^ OR (95% CI)
IQR(lnPFOA)c increase		1,844		106		0.92		1.00 (0.76, 1.32)		3,828		224		1.06		1.02 (0.86, 1.21)
100-ng/mL increase		1,844		106		1.01		1.20 (0.86, 1.68)		3,828		224		1.06		1.06 (0.86, 1.31)
< 40th percentile (1.0 to < 6.1 ng/mLd)		504		35		1.0		1.0		1,527		82		1.0		1.0
40th to < 60th percentile (6.1 to < 10.2 ng/mL)		330		15		0.9		0.9 (0.4, 2.0)		764		48		1.2		1.0 (0.7, 1.6)
60th to < 80th percentile (10.2 to < 21.0 ng/mL)		487		31		1.1		1.0 (0.5, 1.7)		759		51		1.1		1.0 (0.6, 1.5)
≥ 80th percentile (21.0 to 717.6 ng/mL)		523		25		0.7		0.8 (0.5, 1.5)		778		43		1.1		1.0 (0.7, 1.5)
aWest Virginia only. bAdjusted for maternal age, education, parity, smoking status, exposure year, state of residence. cEffect estimates represent the change in outcome for a shift from the 25th percentile to the 75th percentile in estimated PFOA serum levels [IQR(lnPFOA) = 1.50]. dThe category boundaries are from the first imputed data set.																

**Table 3:** Study I: PFOA and pregnancy outcome based on birth records: association of PFOA with preterm birth, Mid-Ohio Valley, 1990–2004.

< 37 weeks gestation					< 32 weeks gestation				
Estimated PFOA	Term births (n)	Cases (n)	Crude OR	Adjusted^a^ OR (95% CI)	Cases (n)	Crude OR	Adjusted^a^ OR (95% CI)
IQR(lnPFOA)b increase		3,695		3,613		0.99		1.02 (0.96, 1.08)		491		0.95		0.98 (0.86, 1.11)
100-ng/mL increase		3,695		3,613		1.01		1.02 (0.94, 1.10)		491		0.88		0.90 (0.74, 1.10)
< 40th percentile (1.0 to < 6.1 ng/mLc)		1,460		1,467		1.0		1.0		197		1.0		1.0
40th to < 60th percentile (6.1 to < 10.2 ng/mL)		755		720		1.0		1.0 (0.8, 1.1)		108		1.0		1.0 (0.7, 1.4)
60th to < 80th percentile (10.2 to < 21.0 ng/mL)		733		714		1.0		1.0 (0.9, 1.2)		95		1.0		1.1 (0.8, 1.4)
≥ 80th percentile (21.0 to 717.6 ng/mL)		747		712		1.0		1.0 (0.9, 1.2)		91		0.9		1.0 (0.7, 1.3)
aAdjusted for maternal age, education, parity, smoking status, exposure year, state of residence. bEffect estimates represent the change in outcome for a shift from the 25th percentile to the 75th percentile in estimated PFOA serum levels [IQR(lnPFOA) = 1.50]. cThe category boundaries are from the first imputed data set.														

PFOA was not related to risk of term low birth weight, with some suggestion of a reduced risk of SGA with higher exposure but no gradient across quintiles (Table 4). Birth weight among term births was largely unrelated to estimated PFOA (Table 4). There was a suggestion of a modest decrease in birth weight with higher exposure based on continuous exposure measures {somewhat stronger for male births [see Supplemental Material, Table 1 (http://dx.doi.org/10.1289/ehp.1104752)]}, but no monotonic gradient across quintiles. In the sensitivity analysis restricted to the 66% of births with street-level geocodes and complete covariate information (see Supplemental Material, Tables 2–4), results were largely unchanged except for generating weak positive associations with both pregnancy-induced hypertension and late preterm births.

**Table 4:** Study I: PFOA and pregnancy outcome based on birth records: association of PFOA with indicators of fetal growth, Mid-Ohio Valley, 1990–2004.

Term low birth weight							Term SGA							Change in term birth weight (g)				
Estimated PFOA	Term births ≥ 2,500 g (n)	Cases (n)	Crude OR	Adjusted^a^ OR (95% CI)	Term AGA (n)	Cases (n)	Crude OR	Adjusted^a^ OR (95% CI)	Cases (n)	Crude difference	Adjusted^a^ difference (95% CI)
IQR(lnPFOA)b increase		3,616		918		0.96		1.02 (0.92, 1.13)		2,990		353		0.85		0.91 (0.78, 1.06)		4,534		3.20		–10.72 (–32.26, 10.82)
100-ng/mL increase		3,616		918		0.91		1.00 (0.86, 1.15)		2,990		353		0.78		0.86 (0.67, 1.11)		4,534		6.44		–14.80 (–43.28, 13.68)
< 40th percentile (1.0 to < 6.1 ng/mL)c		1,420		378		1.0		1.0		1,172		148		1.0		1.0		1,798		0		0 (referent)
40th to < 60th percentile (6.1 to < 10.2 ng/mL)		741		176		0.9		0.9 (0.7, 1.2)		600		81		1.0		1.0 (0.7, 1.4)		917		21.3		22.8 (–32.9, 78.5)
60th to < 80th percentile (10.2 to < 21.0 ng/mL)		717		188		1.0		1.0 (0.8, 1.3)		596		69		1.0		1.0 (0.7, 1.5)		905		–9.6		2.3 (–50.3, 54.8)
≥ 80th percentile (21.0 to 717.6 ng/mL)		738		176		0.9		1.0 (0.8, 1.3)		622		55		0.7		0.8 (0.6, 1.2)		914		10.9		–9.5 (–58.4, 39.4)
AGA, appropriate for gestational age. aAdjusted for maternal age, education, parity, smoking status, exposure year, state of residence, gestational age (Term birth weight analysis only). bEffect estimates represent the change in outcome for a shift from the 25th percentile to the 75th percentile in estimated PFOA serum levels [IQR(lnPFOA) = 1.50]. cThe category boundaries are from the first imputed data set.																						

## Study II: PFOA and Pregnancy Outcome Based on Birth Records Linked to the C8 Health Project

### Methods

The C8 Health Project enrolled > 69,000 residents of the mid-Ohio Valley who were part of a class action lawsuit, administered a questionnaire eliciting health and residential history information, and collected blood to measure serum levels of perfluoroalkyl acids and clinical health markers. Geocoding had already been attempted for every residential address in the C8 Health Project (Shin et al. 2011b).

We sought to link births from 1990–2004 birth certificates in a 13-county region in West Virginia (Cabell, Jackson, Mason, Pleasants, Putnam, Ritchie, Wirt, and Wood counties) and Ohio (Athens, Gallie, Meigs, Morgan, and Washington counties) to self-reported births in the C8 Health Project. This 13-county region encompasses the 5 counties included in study I. We considered a match to be accurate based on consistency between the birth certificate and C8 Health Project information for one of three combinations: *a*) mother’s county and state of residence + mother’s date of birth + month and year of infant’s birth + infant’s sex; *b*) mother’s county and state of residence + mother’s age + mother’s first name, middle initial, last name + month and year of infant’s birth + infant sex; or *c*) mother’s county and state of residence + mother’s address + mother’s age + month and year of infant’s birth + infant sex. This process yielded 3,323 matched births. We extended the matching through the West Virginia Birth Score project, a program of the West Virginia Department of Health (2012) to identify infants at risk of developmental problems. Using the mother’s Social Security number as well as other birth certificate items, Birth Score was able to identify an additional 1,224 matched births through 2004, yielding a total of 4,547 birth certificates linked to the C8 Health Project for analysis.

We used the same environmental/pharmacokinetic models from study I to generate annual exposure estimates, except that the link to the C8 Health Project enabled us to incorporate lifetime residential history, thus reducing exposure misclassification (Shin et al. 2011a, 2011b). We examined the model-based exposure estimates, a Bayesian time-dependent calibration in which the measured 2005–2006 serum concentration was used for updating estimates (Savitz et al. 2012), and a traditional calibration method assuming that a higher-than-expected 2005–2006 serum concentration reflects an entire lifetime of higher-than-expected exposure. As in study I, we considered a log-transformed continuous measure, an untransformed continuous measure, and quintiles, aggregating the lowest two quintiles as the referent. Pregnancy outcomes and covariates were defined as described for study I. We used generalized estimating equations with an exchangeable correlation structure to account for correlation across a woman’s multiple pregnancies.

### Results

As in study I, PFOA exposures were higher in the more recent time period, among older and more highly educated mothers, and among nonsmokers; however, in study II exposures were higher for Ohio births than West Virginia births, presumably reflecting the different geographic scope of the two studies (Table 5).

**Table 5:** Study II: PFOA and pregnancy outcome based on birth records linked to the C8 Health Project: characteristics of the study population, Mid-Ohio Valley, 1990–2004 [n (%)].

Maternal characteristic	PIH	Preterm < 37 weeks	Term LBW	SGA	All births	Estimated PFOA among all births (ng/mL) [median (25th, 75th percentiles)]	
Total		250		405		99		362		4,547			13.4 (5.6, 61.2)
Exposure year													
1990–1994		53 (21)		77 (19)		28 (28)		105 (29)		1,296 (29)			7.2 (4.6, 42.8)
1995–1999		110 (44)		142 (35)		31 (31)		121 (33)		1,674 (37)			13.7 (5.9, 65.7)
2000–2004		87 (35)		186 (46)		40 (40)		136 (38)		1,577 (35)			18.3 (7.6, 63.2)
State of residence													
West Virginia		150 (60)		227 (56)		64 (65)		213 (59)		2,735 (60)			10.4 (5.3, 44.6)
Ohio		100 (40)		178 (44)		35 (35)		149 (41)		1,812 (40)			23.9 (6.6, 81.2)
Age (years)													
< 20		32 (13)		51 (13)		19 (19)		66 (18)		541 (12)			8.8 (5.0, 33.7)
20–24		82 (33)		124 (31)		37 (37)		149 (41)		1,514 (33)			10.8 (5.3, 46.0)
25–29		77 (31)		118 (29)		23 (23)		81 (22)		1,320 (29)			15.6 (5.8, 73.1)
30–34		46 (18)		79 (20)		18 (18)		51 (14)		855 (19)			19.0 (6.3, 74.6)
≥ 35		13 (5)		33 (8)		2 (2)		15 (4)		317 (7)			23.0 (6.3, 106.1)
Education (years)													
< 12		26 (10)		65 (16)		24 (24)		86 (24)		605 (13)			8.6 (5.1, 23.1)
12		109 (44)		176 (43)		46 (46)		157 (43)		1,970 (43)			11.3 (5.2, 50.3)
13–15		67 (27)		110 (27)		17 (17)		83 (23)		1,309 (29)			18.7 (6.3, 79.6)
≥ 16		48 (19)		54 (13)		12 (12)		36 (10)		663 (15)			22.4 (6.3, 84.2)
Parity													
0		167 (67)		189 (47)		52 (53)		189 (52)		2,007 (44)			11.9 (5.5, 52.8)
1		50 (20)		143 (35)		24 (24)		108 (30)		1,652 (36)			15.6 (5.8, 70.0)
≥ 2		33 (13)		73 (18)		23 (23)		65 (18)		888 (20)			13.2 (5.4, 61.5)
Smoking status													
Smoker		20 (8)		96 (24)		40 (40)		154 (43)		870 (19)			10.2 (5.2, 32.4)
Nonsmoker		230 (92)		309 (76)		59 (60)		208 (57)		3,677 (81)			14.7 (5.7, 68.1)
Abbreviations: PIH: pregnancy-induced hypertension; LBW, low birth weight.													

Pregnancy-induced hypertension showed essentially no association with estimated serum PFOA based on the uncalibrated estimates (Table 6). With Bayesian calibration, the log-transformed and categorical measures suggested a positive association, with an increased risk primarily in the 4th quintile. Traditional calibration showed elevated risk in the 3rd and 4th quintiles, but the continuous exposure measures did not indicate an association.

**Table 6:** Study II: PFOA and pregnancy outcome based on birth records linked to the C8 Health Project: association of PFOA with pregnancy-induced hypertension, Mid-Ohio Valley, 1990–2004.

Estimated PFOA	Live births (n)	Cases (n)	Crude OR	Adjusted^a^ OR (95% CI)
Uncalibrated								
IQR(lnPFOA)b increase		3,905		250		1.08		1.05 (0.85, 1.31)
100-ng/mL increase		3,905		250		0.98		1.01 (0.91, 1.12)
< 40th percentile (3.9 to < 8.9 ng/mL)		1,632		96		1.0		1.0
40th to < 60th percentile (8.9 to < 21.8 ng/mL)		778		52		1.1		1.0 (0.7, 1.4)
60th to < 80th percentile (21.8 to 83.3 ng/mL)		736		52		1.2		1.1 (0.7, 1.5)
≥ 80th percentile (83.3 to 921.3 ng/mL)		759		50		1.1		1.1 (0.8, 1.5)
Bayesian calibration								
IQR(lnPFOA)c increase		3,905		250		1.11		1.13 (0.92, 1.37)
100-ng/mL increase		3,905		250		0.92		0.97 (0.85, 1.11)
< 40th percentile (3.9 to < 8.9 ng/mL)		1,635		88		1.0		1.0
40 to < 60th percentile (8.9 to < 19.6 ng/mL)		757		46		1.1		1.0 (0.7, 1.4)
60 to < 80th percentile (19.6 to 53.1 ng/mL)		735		67		1.7		1.5 (1.1, 2.1)
≥ 80th percentile (53.1 to 1897.0 ng/mL)		778		49		1.2		1.2 (0.8, 1.7)
Traditional calibration								
IQR(lnPFOA)d increase		3,905		250		1.01		1.03 (0.87, 1.22)
100-ng/mL increase		3,905		250		0.83		0.89 (0.75, 1.06)
< 40th percentile (0.05 to < 11.4 ng/mL)		1,628		82		1.0		1.0
40th to < 60th percentile (11.4 to < 21.0 ng/mL)		759		63		1.6		1.5 (1.1, 2.2)
60th to < 80th percentile (21.0 to 49.0 ng/mL)		729		62		1.7		1.5 (1.1, 2.2)
≥ 80th percentile (49.0 to 2468.4 ng/mL)		789		43		1.1		1.1 (0.8, 1.7)
aAdjusted for maternal age, education, parity, smoking status, exposure year, state of residence. bEffect estimates represent the change in outcome for a shift from the 25th percentile to the 75th percentile in estimated PFOA serum levels [IQR(lnPFOA) = 2.39]. cEffect estimates represent the change in outcome for a shift from the 25th percentile to the 75th percentile in estimated PFOA serum levels [IQR(lnPFOA) = 1.92]. dEffect estimates represent the change in outcome for a shift from the 25th percentile to the 75th percentile in estimated PFOA serum levels [IQR(lnPFOA) = 1.61].								

Preterm birth < 37 weeks was weakly related to PFOA levels based on continuous measures in the uncalibrated analyses, but with no gradient across quintiles (Table 7). Bayesian calibration yielded a similar pattern and traditional calibration yielded no association. Preterm birth < 32 weeks, with only 40 cases, showed some suggestion of an association without calibration, enhanced somewhat with traditional calibration, and markedly strengthened with Bayesian calibration.

**Table 7:** Study II: PFOA and pregnancy outcome based on birth records linked to the C8 Health Project: association of PFOA with preterm birth, Mid-Ohio Valley, 1990–2004.

< 37 weeks gestation					< 32 weeks gestation				
Estimated PFOA	Term births (n)	Cases (n)	Crude OR	Adjusted^a^ OR (95% CI)	Cases (n)	Crude OR	Adjusted^a^ OR (95% CI)
Uncalibrated														
IQR(lnPFOA)b increase		4,142		405		1.13		1.09 (0.91, 1.32)		40		1.16		1.29 (0.74, 2.23)
100-ng/mL increase		4,142		405		1.06		1.09 (1.00, 1.18)		40		1.00		1.10 (0.86, 1.40)
< 40th percentile (3.9 to < 8.9 ng/mL)		1,669		150		1.0		1.0		13		1.0		1.0
40th to < 60th percentile (8.9 to < 21.8 ng/mL)		810		99		1.4		1.2 (0.9, 1.5)		12		1.9		1.5 (0.7, 3.5)
60th to < 80th percentile (21.8 to 83.3 ng/mL)		841		68		0.9		0.8 (0.6, 1.1)		7		1.1		0.9 (0.4, 2.4)
≥ 80th percentile (83.3 to 921.3 ng/mL)		822		88		1.2		1.2 (0.9, 1.6)		8		1.2		1.4 (0.5, 3.6)
Bayesian calibration														
IQR(lnPFOA)c increase		4,142		405		1.09		1.10 (0.92, 1.31)		40		1.33		1.67 (1.03, 2.70)
100-ng/mL increase		4,142		405		1.06		1.12 (1.02, 1.23)		40		1.06		1.25 (1.02, 1.52)
< 40th percentile (3.9 to < 8.9 ng/mL)		1,666		153		1.0		1.0		10		1.0		1.0
40th to < 60th percentile (8.9 to < 19.6 ng/mL)		820		89		1.2		1.0 (0.8, 1.3)		9		1.8		1.5 (0.6, 4.1)
60th to < 80th percentile (19.6 to 53.1 ng/mL)		827		82		1.1		0.9 (0.7, 1.3)		12		2.4		2.3 (1.0, 5.5)
≥ 80th percentile (53.1 to 1897.0 ng/mL)		829		81		1.1		1.0 (0.8, 1.4)		9		1.8		2.3 (0.9, 5.7)
Traditional calibration														
IQR(lnPFOA)d increase		4,142		405		0.98		0.95 (0.82, 1.11)		40		1.17		1.33 (0.86, 2.07)
100-ng/mL increase		4,142		405		0.99		1.03 (0.94, 1.13)		40		1.01		1.13 (0.97, 1.32)
< 40th percentile (0.05 to < 11.4 ng/mL)		1,649		170		1.0		1.0		13		1.0		1.0
40th to < 60th percentile (11.4 to < 21.0 ng/mL)		831		78		0.9		0.8 (0.6, 1.1)		7		1.0		1.0 (0.4, 2.6)
60th to < 80th percentile (21.0 to 49.0 ng/mL)		825		84		1.0		0.9 (0.7, 1.1)		14		2.1		2.1 (1.0, 4.5)
≥ 80th percentile (49.0 to 2468.4 ng/mL)		837		73		0.9		0.9 (0.6, 1.2)		6		0.9		1.2 (0.4, 3.1)
aAdjusted for maternal age, education, parity, smoking status, exposure year, state of residence. bEffect estimates represent the change in outcome for a shift from the 25th percentile to the 75th percentile in estimated PFOA serum levels [IQR(lnPFOA) = 2.39]. cEffect estimates represent the change in outcome for a shift from the 25th percentile to the 75th percentile in estimated PFOA serum levels [IQR(lnPFOA) = 1.92]. dEffect estimates represent the change in outcome for a shift from the 25th percentile to the 75th percentile in estimated PFOA serum levels [IQR(lnPFOA) = 1.61].														

Term low birth weight was largely unrelated to PFOA based on exposure measures without calibration, except for an isolated increased risk in the 4th quintile (Table 8). With both calibration methods, the log-transformed continuous measure showed a stronger association than the untransformed continuous measure. There were also irregular findings across quintiles, but none suggestive of a monotonic gradient. Term SGA was weakly associated with increasing PFOA levels based on continuous and categorical exposures across all exposure estimates (Table 8). The continuous birth weight analysis yielded some support for an association (Table 8), with a predicted decrement of 22 g for an interquartile shift in exposure (95% CI: –46, 2.1 g), and decrements of 25 g (95% CI: –64, 13 g) and 33 g (95% CI: –73, 6.5 g) in the fourth and fifth quintiles of exposure based on the uncalibrated exposure estimates. This pattern was similar for the Bayesian calibrated estimates and somewhat reduced with traditional calibration. No sex differences were apparent [see Supplemental Material, Table 5 (http://dx.doi.org/10.1289/ehp.1104752)].

**Table 8:** Study II: PFOA and pregnancy outcome based on birth records linked to the C8 Health Project: association of PFOA with indicators of fetal growth, Mid-Ohio Valley, 1990–2004.

Term low birth weight					Term SGA						Change in term birth weight (g)		
Estimated PFOA	Term births ≥ 2,500 g (n)	Cases (n)	Crude OR	Adjusted^a^ OR (95% CI)	Term, AGA (n)	Cases (n)	Crude OR	Adjusted^a^ OR (95% CI)					
									Cases (n)	Crude difference	Adjusted^a^ difference (95% CI)
Uncalibrated																
IQR(lnPFOA)b increase	4,043	99		0.87	1.04 (0.75,1.44)		3,375		362		1.02	1.18 (0.97, 1.43)		4,142	0.97	–21.89 (–45.91, 2.13)
100-ng/mL increase	4,043	99		0.93	1.00 (0.82,1.21)		3,375		362		1.01	1.07 (0.98, 1.17)		4,142	4.27	–9.14 (–20.30, 2.02)
< 40th percentile (3.9 to < 8.9 ng/mL)	1,629	40		1.0	1.0		1,356		144		1.0	1.0		1,669	0	0 (referent)
40th to < 60th percentile (8.9 to < 21.8 ng/mL)	791	19		0.9	0.9 (0.5, 1.7)		659		72		1.0	1.0 (0.7, 1.4)		810	–19.9	–3.8 (–40.4, 32.8)
60th to < 80th percentile (21.8 to 83.3 ng/mL)	814	27		1.4	1.6 (1.0, 2.8)		689		76		1.0	1.1 (0.8, 1.6)		841	–30.4	–25.4 (–63.7, 12.9)
≥ 80th percentile (83.3 to 921.3 ng/mL)	809	13		0.7	0.9 (0.5, 1.7)		671		70		1.0	1.3 (0.9, 1.7)		822	4.9	–33.3 (–73.1, 6.5)
Bayesian calibration																
IQR(lnPFOA)c increase	4,043	99		0.97	1.16 (0.86,1.58)		3,375		362		1.03	1.19 (1.00, 1.43)		4,142	7.78	–21.51 (–43.62, 0.61)
100-ng/mL increase	4,043	99		0.93	1.04 (0.85,1.27)		3,375		362		0.98	1.06 (0.97, 1.16)		4,142	3.73	–18.55 (–31.31, –5.80)
< 40th percentile (3.9 to < 8.9 ng/mL)	1,624	42		1.0	1.0		1,358		148		1.0	1.0		1,666	0	0 (referent)
40th to < 60th percentile (8.9 to < 19.6 ng/mL)	803	17		0.8	0.8 (0.4, 1.5)		676		68		0.9	0.9 (0.7, 1.3)		820	–15.8	10.8 (–24.9, 46.5)
60th to < 80th percentile (19.6 to 53.1 ng/mL)	803	24		1.1	1.3 (0.7, 2.2)		664		74		1.0	1.1 (0.8, 1.5)		827	–9.0	–11.0 (–49.8, 27.8)
≥ 80th percentile (53.1 to 1897.0 ng/mL)	813	16		0.8	1.0 (0.6, 1.9)		677		72		1.0	1.3 (0.9, 1.7)		829	8.7	–32.3 (–71.5, 6.8)
Traditional calibration																
IQR(lnPFOA)d increase	4,043	99		1.16	1.33 (1.04,1.69)		3,375		362		1.05	1.17 (1.00, 1.36)		4,142	2.45	–16.90 (–34.89, 1.08)
100-ng/mL increase	4,043	99		1.00	1.07 (0.96,1.18)		3,375		362		1.03	1.08 (1.01, 1.16)		4,142	4.54	–12.76 (–26.08, 0.57)
< 40th percentile (0.05 to < 11.4 ng/mL)	1,614	35		1.0	1.0		1,351		147		1.0	1.0		1,649	0	0 (referent)
40th to < 60th percentile (11.4 to < 21.0 ng/mL)	817	14		0.8	0.8 (0.4, 1.5)		686		70		0.9	1.0 (0.7, 1.3)		831	–9.2	4.2 (–31.2, 39.6)
60th to < 80th percentile (21.0 to 49.0 ng/mL)	793	32		1.8	2.2 (1.3, 3.6)		659		72		1.0	1.1 (0.8, 1.5)		825	1.1	1.8 (–37.7, 41.4)
≥ 80th percentile (49.0 to 2468.4 ng/mL)	819	18		1.1	1.4 (0.8, 2.5)		679		73		1.0	1.2 (0.9, 1.7)		837	22.7	–21.2 (–59.6, 17.2)
AGA, appropriate for gestational age. aAdjusted for maternal age, education, parity, smoking status, exposure year, state of residence, gestational age (term birth weight analysis only). bEffect estimates represent the change in outcome for a shift from the 25th percentile to the 75th percentile in estimated PFOA serum levels [IQR(lnPFOA) = 2.39]. cEffect estimates represent the change in outcome for a shift from the 25th percentile to the 75th percentile in estimated PFOA serum levels [IQR(lnPFOA) = 1.92]. dEffect estimates represent the change in outcome for a shift from the 25th percentile to the 75th percentile in estimated PFOA serum levels [IQR(lnPFOA) = 1.6].																

## Discussion

In study I, PFOA and Pregnancy Outcome Based on Birth Records, we found little indication of an association between PFOA and stillbirth, pregnancy-induced hypertension, preterm birth, or indicators of fetal growth. These analyses had the advantage of relative freedom from selection bias and large study size, but exposure was estimated based only on the residential address reported on the birth certificate rather than a lifetime residential history. Study II, PFOA and Pregnancy Outcome Based on Birth Records Linked to the C8 Health Project, yielded sporadic positive associations but little overall support for an association with pregnancy-induced hypertension or preterm birth< 37 weeks, modest support for an association with indicators of fetal growth restriction, and strong but imprecise indications of an association with preterm birth < 32 weeks. These analyses incorporated better estimates of exposure by using lifetime residential history, but a smaller sample size and the potential for selection bias from incomplete matching of birth certificates to the C8 Health Project.

Study I and study II differ in several ways, including comprehensiveness and precision, but exposure assignment is probably most influential: Study II had residential history information for reconstructing exposure, and study I did not. Among the participants in both studies (*n* = 626), the Spearman rank order correlation coefficient between the exposure estimate based on reported lifetime residential history versus just the maternal residence on the birth certificate was 0.64, reflecting a modest correlation and a clear loss of accuracy in study I.

There is some overlap between the births included in the two studies given the shared geographic scope. More than 70% of preterm and term low birth weight cases in study II were also included in study I because of the comprehensive assessment of these case groups in study I. Fewer than 8% of all study I births, however, were included in study II.

Integrating these findings into the literature, there are three previous studies in this geographic area (Nolan et al. 2009; Savitz et al. 2012; Stein et al. 2009) as well as other studies of PFOA and birth outcomes (Apelberg et al. 2007; Fei et al. 2007; Hamm et al. 2010). The study by Nolan et al. (2009) addressed births in 2003–2005 in Washington County, Ohio, comparing birth weight and preterm birth risk among residents of ZIP codes with varying levels of PFOA in the water.

Stillbirth was examined in our previous analysis of C8 Health Project participants (Savitz et al. 2012) and neither Savitz et al. (2012) nor the present study suggested an association with PFOA. Preeclampsia was weakly associated with PFOA exposure in other analyses of this population (Savitz et al. 2012; Stein et al. 2009). Study I showed essentially no association between PFOA and pregnancy-induced hypertension, whereas study II provided modest support, primarily with calibrated exposure estimates. Among all pregnancy outcome measures, pregnancy-induced hypertension is the most vulnerable to inconsistent definitions and inaccurate reporting. The C8 Health Project addresses self-reported “preeclampsia” (Savitz et al. 2012; Stein et al. 2009) and birth certificates code “pregnancy-induced hypertension,” although there is likely to be misclassification in both data sources. Pregnancy-induced hypertension refers to elevated blood pressure with or without protein in the urine; preeclampsia specifically requires proteinuria. When compared with medical records, birth certificates yield sensitivity and positive predictive values < 50% (DiGiuseppe et al. 2002; Lydon-Rochelle et al. 2005). These largely negative findings should be tempered by recognition of substantial outcome misclassification.

Evaluation of preterm birth in previous studies (Fei et al. 2007; Hamm et al. 2010) was hampered by small numbers of cases and yielded little evidence of an association. Previous reports on preterm birth in this study area, whether based on serum measures in recent pregnancies (Stein et al. 2009), historical exposure estimates among larger numbers of C8 Health Project pregnancies (Savitz et al. 2012), or geographic analyses of exposed ZIP codes (Nolan et al. 2009) are consistent in showing an absence of association. Study I likewise showed no evidence of an association, but the findings in study II are more ambiguous. Preterm birth < 37 weeks had at most modest suggestions of an association, whereas preterm birth < 32 weeks generated large and imprecise elevations in risk, markedly strengthened by Bayesian calibration to estimate exposure. Although the size of the associations warrant attention, the role of random error and instability across exposure measures must also be taken into account.

Previous analyses of reduced birth weight have focused largely on continuous birth weight measures rather than on low birth weight or SGA. Studies of other populations have had limited ability to precisely examine extreme levels of birth weight and have found no support for an association with PFOA (Fei et al. 2007; Hamm et al. 2010). Two previous studies provided support for an association between continuous measures of PFOA and birth weight (Apelberg et al. 2007; Fei et al. 2007), and two smaller studies provided less precise statistical indications of modest reductions in birth weight with increasing levels of PFOA (Hamm et al. 2010; Washino et al. 2009). Previous analyses in the Mid-Ohio Valley showed little indication of an effect of PFOA on fetal growth (Nolan et al. 2009; Savitz et al. 2012; Stein et al. 2009), but studies based on just the C8 Health Project (Savitz et al. 2012; Stein et al. 2009) were unable to examine a continuous measure of birth weight. In study I, there was some indication of decrements in birth weight associated with increasing PFOA levels, particularly for male births in the categorical analysis. In study II, all growth indicators—term low birth weight, term SGA, and a continuous measure of birth weight—showed small, inconsistent suggestions of a possible association, which is comparable with the previous reports.

There are a number of issues to consider with regard to possible decrements in birth weight associated with PFOA exposure. First, the studies of typical levels of PFOA (Apelberg et al. 2007; Fei et al. 2007) yield associations that are similar to our effect sizes even though we examined a much greater range of exposure. If there is a causal effect in which the impact is linear at both very low and very high exposures, we would have expected the larger absolute exposure contrasts in the present study to yield stronger associations than those from studies of a narrower exposure range. Second, the previous studies examining typical serum levels of PFOA during pregnancy were susceptible to distortion by relying on exposure measures that reflect the uptake, metabolism, and excretion of PFOA, and may therefore share some physiologic determinants of birth weight (Longnecker 2006). In contrast, exposure assignments in the present study (except for the calibrated estimates in study II) were based on distinctive sources of environmental contamination and were unaffected by physiologic differences in PFOA metabolism. Third, the physiologic or clinical relevance of these small shifts in birth weight is uncertain, possibly serving as a marker of subtle toxicity, but not of direct importance to health. Not only are the implications of low birth weight subject to varying interpretations (Wilcox 2001), but shifts within the normal range, which drives the association because most births are within the normal range, are even more questionable as markers of adverse impacts on health.

The limitations in these data warrant comment, with most issues applicable to both studies. Birth certificates are a useful, if imperfect, source of data on pregnancy outcome: Birth weight is generally accurate, more so than gestational age (DiGiuseppe et al. 2002), with pregnancy-induced hypertension the most fallible outcome. Information on potential confounders was limited to basic demographic information and smoking, though the spatial manner in which the exposure was distributed makes strong confounding by socioeconomic or behavioral factors unlikely. Even the exposure estimates based on full residential histories in study II are subject to error, given the inability to predict with precision the levels of contaminants in drinking water at a given place and time, individual variability in water use and PFOA pharmacokinetics, and inaccuracies in dates and locations of self-reported residences. The magnitude of these errors is exacerbated in study I because of the absence of longitudinal information on residential history.

Overall, we found little support for PFOA having adverse effects on pregnancy-induced hypertension or preterm birth in the aggregate; modest and inconsistent evidence of reduced fetal growth; and large, imprecise indications of an association with early preterm birth. The irregularity across exposure indices and susceptibility to inconsistent variation based on calibration tempers the suggestions of possible causal effects on these pregnancy outcomes. The potential association of PFOA with small decrements in birth weight is of unknown significance, but is perhaps the most readily amenable to more refined studies for continued investigation, with studies of preterm birth < 32 weeks, a rare outcome, requiring nested case–control studies based on stored serum for exposure assessment. If an impact on birth weight is causal, which is yet to be established, it reflects a biological process that is worth elucidating even if not of direct health concern.

## Supplemental Material

(131 KB) PDF
