# Supplementary material for: Relationship of Perfluorooctanoic Acid Exposure to Pregnancy Outcome Based on Birth Records in the Mid-Ohio Valley
Source: Environ Health Perspect. 2012 Mar 26;120(8):1201–7. doi: 10.1289/ehp.1104752 (PMC3440089; doi:10.1289/ehp.1104752)
Supplement: (131 KB) PDF [file ehp.1104752.s001.pdf]

Relationship of Perfluorooctanoic Acid Exposure to Pregnancy Outcome Based on Birth Records in the Mid-Ohio Valley

David A. Savitz, Cheryl R. Stein, Beth Elston, Gregory A. Wellenius, Scott M. Bartell, Hyeong-Moo Shin, Veronica M. Vieira, Tony Fletcher

Table of Contents

Page 2 -- Supplemental Material, Table 1. Study I: PFOA and Pregnancy Outcome Based on Birth Records. Association of PFOA Serum Concentration with Term Birthweight, Stratified by Sex, Mid-Ohio Valley, 1990-2004

Page 3 -- Supplemental Material, Table 2. Study I: PFOA and Pregnancy Outcome Based on Birth Records with Street-Level Geocodes. Association PFOA with Stillbirth and Pregnancy-induced Hypertension, Mid-Ohio Valley, 1990-2004

Page 4 -- Supplemental Material, Table 3. Study I: PFOA and Pregnancy Outcome Based on Birth Records with Street-Level Geocodes. Association of PFOA with Preterm Birth, Mid-Ohio Valley, 1990-2004

Page 5 -- Supplemental Material, Table 4. Study I: PFOA and Pregnancy Outcome Based on Birth Records with Street-Level Geocodes. Association of PFOA with Indicators of Fetal Growth, Mid-Ohio Valley, 1990-2004

Page 6 -- Supplemental Material, Table 5. Study II: PFOA and Pregnancy Outcome Based on Birth Records Linked to the C8 Health Project. Association of PFOA with Term Birthweight, Stratified by Sex, Mid-Ohio Valley, 1990-2004

Supplemental Material, Table 1. Study I: PFOA and Pregnancy Outcome Based on Birth Records. Association of PFOA Serum Concentration with Term Birthweight, Stratified by Sex, Mid-Ohio Valley, 1990-2004

Estimated PFOA	Females			Males		
		Change in birthweight (g)			Change in birthweight (g)	
	n	Crude	Adjusted[a] (95% CI)	n	Crude	Adjusted[a] (95% CI)
IQR(lnPFOA)[b] increase	2305	16.33	2.68 (-26.12, 31.47)	2229	-7.43	-20.41 (-51.64, 10.81)
100 ng/mL increase	2305	5.17	-7.88 (-45.57, 29.80)	2229	11.45	-16.50 (-59.03, 26.03)
<40th percentile 1.0 - <6.1 ng/mL[c]	888	0	0 (referent)	910	0	0 (referent)
40 – <60th percentile 6.1 - <10.2 ng/mL	478	39.9	41.5 (-27.8, 110.8)	439	11.0	16.0 (-61.0, 93.1)
60 – <80th percentile 10.2 - <21.0 ng/mL	468	13.0	22.1 (-46.2, 90.3)	437	-18.9	-5.9 (-83.0, 71.1)
≥80th percentile 21.0 - 717.6 ng/mL	471	39.3	17.1 (-48.9, 83.2)	443	-13.1	-30.6 (-100.9, 39.7)

[a] adjusted for maternal age, education, parity, smoking status, exposure year, state of residence, gestational age
[b] Effect estimates represent the change in outcome for a shift from the 25th percentile to the 75th percentile in estimated PFOA serum levels (IQR(lnPFOA) = 1.50)
[c] The category boundaries are from the 1st imputed dataset

Supplemental Material, Table 2. Study I: PFOA and Pregnancy Outcome Based on Birth Records with Street-Level Geocodes. Association PFOA with Stillbirth and Pregnancy-induced Hypertension, Mid-Ohio Valley, 1990-2004

	Stillbirth				Pregnancy-induced Hypertension			
Estimated PFOA	Live Births[b] n	Case n	Crude OR	Adjusted[a] OR (95% CI)	Live Births n	Case n	Crude OR	Adjusted[a] OR (95% CI)
IQR(lnPFOA)[c] increase	1514	68	0.93	1.00 (0.69, 1.41)	2531	157	1.12	1.13 (0.91, 1.38)
100 ng/mL increase	1514	68	0.93	1.05 (0.64, 1.54)	2531	157	1.10	1.14 (0.90, 1.39)
<40th percentile<br>3.9 – <6.1 ng/mL	420	23	1.0	1.0	1019	56	1.0	1.0
40 – <60th percentile<br>6.1 – <11.4 ng/mL	319	9	0.5	0.5 (0.2, 1.1)	533	35	1.2	1.1 (0.7, 1.6)
60 – <80th percentile<br>11.4 – 23.1 ng/mL	394	22	1.0	0.9 (0.5, 1.6)	489	30	1.1	1.0 (0.6, 1.6)
≥80th percentile<br>23.1 – 602.2 ng/mL	381	14	0.7	0.8 (0.4, 1.6)	490	36	1.3	1.3 (0.8, 2.1)

[a] adjusted for maternal age, education, parity, smoking status, exposure year, state of residence
[b] West Virginia only
[c] Effect estimates represent the change in outcome for a shift from the 25th percentile to the 75th percentile in estimated PFOA serum levels (IQR(lnPFOA) = 1.37)

Supplemental Material, Table 3. Study I: PFOA and Pregnancy Outcome Based on Birth Records with Street-Level Geocodes. Association of PFOA with Preterm Birth, Mid-Ohio Valley, 1990-2004

Estimated PFOA	Term Births n	<37 weeks gestation Case n	Crude OR	Adjusted[a] OR (95% CI)	<32 weeks gestation Case n	Crude OR	Adjusted[a] OR (95% CI)	32 - <37 weeks gestation Case n	Crude OR	Adjusted[a] OR (95% CI)
IQR(lnPFOA)[b] increase	2456	2292	1.04	1.07 (0.99, 1.15)	306	0.97	1.05 (0.89, 1.23)	1986	1.05	1.07 (1.00, 1.16)
100 ng/mL increase	2456	2292	1.04	1.06 (0.97, 1.15)	306	0.90	0.94 (0.75, 1.14)	1986	1.06	1.07 (0.98, 1.17)
<40th percentile 3.9 – <6.1 ng/mL	984	921	1.0	1.0	124	1.0	1.0	797	1.0	1.0
40 – <60th percentile 6.1 – <11.4 ng/mL	523	438	0.9	0.9 (0.8, 1.1)	60	0.9	1.0 (0.7, 1.3)	378	0.9	0.9 (0.8, 1.1)
60 – <80th percentile 11.4 – 23.1 ng/mL	471	461	1.0	1.1 (0.9, 1.3)	63	1.1	1.1 (0.8, 1.6)	398	1.0	1.1 (0.9, 1.2)
≥80th percentile 23.1 – 602.2 ng/mL	478	472	1.1	1.1 (1.0, 1.3)	59	1.0	1.1 (0.8, 1.6)	413	1.1	1.1 (1.0, 1.3)

[a] adjusted for maternal age, education, parity, smoking status, exposure year, state of residence
[b] Effect estimates represent the change in outcome for a shift from the 25th percentile to the 75th percentile in estimated PFOA serum levels (IQR(lnPFOA) = 1.37)

Supplemental Material, Table 4. Study I: PFOA and Pregnancy Outcome Based on Birth Records with Street-Level Geocodes. Association of PFOA with Indicators of Fetal Growth, Mid-Ohio Valley, 1990-2004

	Term Low Birthweight				Term Small-for-Gestational-Age				Term Birthweight Change in birthweight (g)		
Estimated PFOA	Term Births ≥2500g n	Case n	Crude OR	Adjusted[a] OR (95% CI)	Term AGA n	Case n	Crude OR	Adjusted[a] OR (95% CI)	n	Crude	Adjusted[a] (95% CI)
IQR(lnPFOA)[b] increase	2404	604	0.98	1.04(0.91,1.17)	2028	225	0.88	0.91 (0.74, 1.11)	3008	-9.17	-13.75 (-39.87, 12.37)
100 ng/mL increase	2404	604	0.91	0.98(0.83,1.13)	2028	225	0.81	0.86 (0.64, 1.09)	3008	2.72	-11.06 (-41.11, 18.99)
<40th percentile<br>3.9 – <6.1 ng/mL	958	245	1.0	1.0	810	94	1.0	1.0	1203	0	0 (referent)
40 – <60th percentile<br>6.1 – <11.4 ng/mL	513	113	0.9	0.9 (0.7, 1.2)	434	47	0.9	0.9 (0.6, 1.4)	626	20.0	24.6 (-29.3, 78.5)
60 – <80th percentile<br>11.4 – 23.1 ng/mL	460	131	1.1	1.1 (0.8, 1.4)	375	49	1.1	1.1 (0.7, 1.6)	591	-26.4	15.8 (-40.3, 71.8)
≥80th percentile<br>23.1 – 602.2 ng/mL	473	115	1.0	1.1 (0.8, 1.4)	409	35	0.7	0.8 (0.5, 1.2)	588	-11.8	-20.4 (-76.1, 35.3)

AGA: Appropriate-for-Gestational- Age
[a] adjusted for maternal age, education, parity, smoking status, exposure year, state of residence, gestational age (birthweight only)
[b] Effect estimates represent the change in outcome for a shift from the 25th percentile to the 75th percentile in estimated PFOA serum levels (IQR(lnPFOA) = 1.37

Supplemental Material, Table 5. Study II: PFOA and Pregnancy Outcome Based on Birth Records Linked to the C8 Health Project. Association of PFOA with Term Birthweight, Stratified by Sex, Mid-Ohio Valley, 1990-2004

	Females			Males		
	Change in birthweight (g)			Change in birthweight (g)		
Estimated PFOA	n	Crude	Adjusted[a] (95% CI)	n	Crude	Adjusted[a] (95% CI)
**Uncalibrated**						
IQR(lnPFOA)[b] increase	2024	-4.00	-26.18 (-58.82, 6.47)	2115	0.95	-24.94 (-57.11, 7.23)
100 ng/mL increase	2024	3.23	-12.52 (-28.70, 3.67)	2115	3.49	-9.10 (-23.24, 5.05)
<40th percentile 3.9 – <8.9 ng/mL	832	0	0 (referent)	836	0	0 (referent)
40 – <60th percentile 8.9 – <21.8 ng/mL	389	-38.0	-18.5 (-66.8, 29.9)	420	-11.8	-0.0 (-55.3, 55.3)
60 – <80th percentile 21.8 – 83.3 ng/mL	416	-56.0	-47.2 (-97.4, 3.0)	425	-9.0	-9.6 (-64.6, 45.3)
≥80th percentile 83.3 – 921.3 ng/mL	387	1.4	-36.1 (-89.5, 17.2)	434	-1.8	-44.0 (-98.9, 10.8)
**Bayesian Calibration**						
IQR(lnPFOA)[c] increase	2024	1.00	-24.54 (-54.16, 5.09)	2115	10.86	-23.45 (-53.64, 6.74)
100 ng/mL increase	2024	-0.20	-22.45 (-41.13, -3.77)	2115	7.42	-15.92 (-32.48, 0.64)
<40th percentile 3.9 – <8.9 ng/mL	837	0	0 (referent)	828	0	0 (referent)
40 – <60th percentile 8.9 – <19.6 ng/mL	386	-31.5	3.8 (-44.4, 52.0)	433	-23.8	-5.9 (-58.0, 46.3)
60 – <80th percentile 19.6 – 53.1 ng/mL	405	3.7	9.9 (-41.2, 61.0)	422	-23.9	-33.1 (-88.3, 22.0)
≥80th percentile 53.1 – 1897.0 ng/mL	396	-8.3	-42.3 (-93.5, 8.9)	432	16.7	-36.9 (-92.3, 18.5)
**Traditional Calibration**						
IQR(lnPFOA)[d] increase	2024	-4.32	-24.32 (-48.75, 0.11)	2115	5.61	-14.93 (-39.29, 9.44)
100 ng/mL increase	2024	8.39	-9.32 (-27.81, 9.17)	2115	-0.86	-18.05 (-33.92, -2.19)
<40th percentile 0.05 – <11.4 ng/mL	822	0	0 (referent)	826	0	0 (referent)
40 – <60th percentile 11.4 – <21.0 ng/mL	399	-23.9	-9.3 (-56.9, 38.3)	431	-5.7	6.7 (-44.6, 57.9)
60 – <80th percentile 21.0 – 49.0 ng/mL	411	-11.4	-11.2 (-61.8, 39.5)	414	8.6	11.7 (-46.1, 69.5)
≥80th percentile 49.0 – 2468.4 ng/mL	392	12.6	-30.9 (-82.4, 20.7)	444	19.8	-31.1 (-84.2, 21.9)

[a] adjusted for maternal age, education, parity, smoking status, exposure year, state of residence, gestational age

[b] Effect estimates represent the change in outcome for a shift from the 25th percentile to the 75th percentile in estimated PFOA serum levels (IQR(lnPFOA) = 2.39)

[c] Effect estimates represent the change in outcome for a shift from the 25th percentile to the 75th percentile in estimated PFOA serum levels (IQR(lnPFOA) = 1.92)

[d] Effect estimates represent the change in outcome for a shift from the 25th percentile to the 75th percentile in estimated PFOA serum levels (IQR(lnPFOA) = 1.61)
